# Pretreatments to bonding on enamel and dentin disorders: a systematic review

**DOI:** 10.1038/s41432-024-01037-z

**Published:** 2024-07-23

**Authors:** Jeanne Voinot, Maxime Bedez

**Affiliations:** Univ. Lille, CHU Lille, Odontologie, F-59000 Lille, France

**Keywords:** Dental diseases, Restorative dentistry

## Abstract

**Introduction:**

This systematic review focuses on structural anomalies of enamel and dentin such as fluorosis (F), molar-incisor hypomineralization (MIH), amelogenesis imperfecta (AI), dentinogenesis imperfecta (DI), osteogenesis imperfecta (OI), and X-linked hypophosphatemia (XLH). These pathologies affect up to 31% of the population, posing challenges in the adhesion of direct restorations. The primary objective of this analysis is to examine the survival rate and/or bonding resistance of direct restorations on tissues affected by enamel and dentin disorders in humans. We aim to provide precise clinical recommendations for dentists to choose the appropriate bonding pretreatment for various enamel and dentin disorders.

**Methods:**

We systematically searched the medical literature to identify abstracts of interest indexed between 1993 and May 2024, from 4 databases (PubMed, PMC-PubMed, Web of Science and Cochrane Library). The results are reported following the PRISMA statement. The GRADE approach was used to assess the risk of bias.

**Results:**

The two authors included 27 studies out of the 600 identified. The data extracted from these studies are highly heterogeneous, and the mentioned bonding protocols are all different. 15 articles pertain to F, 7 to MIH, 5 to AI, and none focus on HSPM, dentin disorders and XLH. We identified 12 pretreatments of enamel or dentin (7 for F, 2 for MIH, and 3 for AI), including 3 strong recommendations. Additional studies are necessary to confirm the effectiveness of certain pretreatments. In developmental enamel defects, the main recommendation we collected is to bond on enamel with an etch-and-rinse technique on F and MIH. Considerations with lower strength of evidence include a deproteinization step on enamel on F, MIH and AI and modifications of the etching parameters on F and AI.

The research strategy was registered on the Prospero platform (CRD42023447502).

Key points

- We used PRISMA and GRADE to identify effective bonding protocols for treating developmental enamel and dentin defects.
- 27 peer-reviewed studies provide comprehensive coverage of the effects of bonding protocols on molar-incisor hypomineralization, fluorosis, and amelogenesis imperfecta.
- Our study is centered on practical applications, specifically targeting improvements in clinical dental practices for treating dental tissue pathologies.

## Introduction

### Aim of these guidelines and specific objectives

Numerous authors have conducted investigations regarding the modification of the pretreatment step to optimize bonding for affected tissues by structural disorders. Studied parameters vary across studies and the specific conditions considered, as summarized in some systematic reviews^1–4^. However, none of these reviews provide practical recommendations encompassing all the previously mentioned pathologies.

The objective of these clinical recommendations is to offer dentists pretreatments tailored to enamel and dentin disorders—including fluorosis (F), hypomineralized second primary molars (HSPM), molar-incisor hypomineralization (MIH), amelogenesis imperfecta (AI), dentinogenesis imperfecta (DI), osteogenesis imperfecta (OI) and X-linked hypophosphatemia (XLH)—in order to enhance the shear bond strength (SBS), microtensile bond strength (µTBS) or survival rate (SR) of direct restorations.

### Description of the health problem

Various diseases affect the mineralized tissues of the tooth, such as fluorosis, HSPM, MIH, AI, DI, OI, and XLH. The characteristics of these enamel and/or dentin structural anomalies are summarized in Table 1, including the affected teeth and tissues, dental clinical aspects, and the prevalence of each condition^4–24^. The classification by de La Dure-Molla is the most recent concerning the different types of DI. In this classification, OI is considered as DI-type I according to Shields’ classification^25^. The prevalence of fluorosis depends on the population studied and their geographical location. Here, the prevalence is particularly high because it involves populations from Canada and Africa, where fluoride levels in water are very high. The figures would be lower in other parts of the world.

**Table 1:** Characteristics of the main enamel and dentin disorders.

Disorder	Dentition	Tissue	Clinical aspect	Prevalence
F^5–7^	Both	E	**Thylstrup & Fejerskov Index (TFI from 0 to 9):** mildest to most severe fluorosis, opacities, dyschromia (yellowish & darkbrown) **TFI 0:** Normal translucency	31:100 to 80:100
MIH^8–11^	FPM and permanent incisors	E	Normal E thickness, dyschromia (white, yellow, brown) **Mild:** opacities with PEB **Moderate:** opacities with PEB limited to E **Severe:** PEB with dentin involvement	2,5:100 to 40:100
HSPM^12–15^	SPM	E	Hypomineralization, opacities, PEB, atypical caries/restorations, extractions due to HSPM	6,8:100
AI^16–18^	Both	E	**Hypoplastic:** thin enamel **Hypomature/Hypocalcified:** soft enamel, dyschromia (yellow/brown to red/brown) **Hypomineralized:** soft enamel	1:140,00 to 1:700
DI^4,19,20^	DD or both	D	**Mild:** normal crown, partial pulp chamber obliteration **Moderate:** dyschromia, total pulp chamber obliteration, bulbous crown **Severe:** dyschromia, enlarged pulp chambers with shell teeth appearance	1:8000 to 1:6000
OI^21,22^	DD or both	D	**Normal crown, partial pulp chamber obliteration** **Type I:** mild OI with bone fragility and blue sclerae **Type II:** perinatal, lethal **Type III:** progressive, deforming **Type IV:** normal sclerae and mild deformity	1:30,000 to 1:20,000
XLH^23,24^	PD	D & E	Abscesses, late secondary dentition, enlarged pulp chambers, D hypomineralization	5:100,000

*AI* amelogenesis imperfecta, *D* dentin, *DD* deciduous dentition, *DI* dentinogenesis imperfecta, *E* enamel, *F* fluorosis, *FPM* first permanent molar, *HSPM* hypomineralized second primary molars, *MIH* molar incisor hypomineralization, *OI* osteogenesis imperfecta, *PEB* post-eruptive enamel breakdown, *PD* permanent dentition, *SPM* second primary molars, *TFI* Thylstrup & Fejerskov Index, *XLH* X-linked hypophosphatemia.

Their etiology is not fully understood, but they are primarily of genetic origin, involving the impairment of certain genes in AI, DI, or OI^18,25,26^, of environmental origin (through excessive repetitive fluoride exposure for F)^27^ or of multifactorial origin depending on the duration, strength and timing of occurrence of the etiological factors^2^. This alteration in the structure of dental tissues reduces SBS/µTBS and the SR of direct restorations.

Depending on the type of disorder, permanent dentition (PD) and/or deciduous dentition (DD) may be affected, requiring patient management to restore masticatory and esthetic functions. Numerous case reports have highlighted the effectiveness of direct restorations on these affected tissues, particularly in mixed dentition^28–30^. This provides transitional therapy prior to the placement of indirect restorations in adulthood, and remains a tissue-preserving technique^31^. Direct restorations involve the application of a composite resin in contact with enamel and dentin through bonding. Bonding consists of a dual interface between the adhesive and the dental tissue (enamel or dentin) and between the adhesive and the composite restoration material. There are two main adhesive systems: the “self-etch” adhesive and the “etch-and-rinse” system^32^.

Several studies demonstrate that bonding performance is compromised in cases of enamel or dentin disorders, particularly in case reports of patients with esthetic demands^2,33,34^. In the study by Yaman et al., the µTBS of direct restorations on enamel affected by AI in PD is 40% lower compared to healthy enamel^35^. In Waidyasekera et al.’s study on bonding in F, the SBS is decreased by 40% on affected dentin^36^. In the study by Sönmez et al. the SR on sound enamel in MIH after 24 months is 100%, while it only reaches 80.7% in the affected group^37^. There are no precise numerical data on the alteration of bonding in DI or OI but it is known that there is a clinical difficulty in bonding to these affected tissues^38^.

## Methods

The Preferred Reporting Items for Systematic Reviews and Meta-Analyses (PRISMA Statement) checklist for reporting was followed^39^. The selection and quality assessment of the studies were carried out by authors with no prior publications on the topic of this systematic review. The research strategy was registered on the Prospero platform (CRD42023447502).

### Search strategy and selection criteria

Four databases were used for the search, from January, 1993 to May, 2024: PubMed, PMC-PubMed, Web of Science, and the Cochrane Library (the search strategies are available in Table 2). Published papers in English were selected from the last 30 years up to the present, which represents a period of significant popularization of direct techniques, as well as increased awareness of structural teeth diseases. This study follows the PICO (population, intervention, comparison, outcome) framework, relative to PRISMA statement: the population is adults or children affected by enamel or dentin disorders; the intervention is resin bonding direct restorations; the comparison is done between different protocols, with and without pretreatment; and the outcomes are SR or SBS. The inclusion criteria for articles selection were the evaluation of the SR or/and SBS of resin bonding for direct restorations, on the tooth affected by F, HSPM, MIH, AI, DI, OI, or XLH. Both clinical studies (in vivo) and laboratory studies (in vitro) were included, with a higher strength of evidence attributed to the former. SR and SBS were selected due to their frequent appearance in the literature and their clinical relevance in humans, making them relevant for clinical recommendations.

**Table 2:** Search strategies used on Cochrane Library, PubMed, PMC-PubMed, and Web of Science.

Cochrane Library From 1993 to 2024	(Molar AND (Hypomineralization OR Hypomineralized) OR MIH OR HSPM OR “amelogenesis imperfecta” OR “dentinogenesis imperfecta” OR “osteogenesis imperfecta” OR hypophosphatemia OR "dentin dysplasia" OR hypomineralization) AND ("dental bonding" OR bond* OR etch*) AND (dental OR tooth)
PubMed From 1993 to 2024	((Molar AND (Hypomineralization OR Hypomineralized)) OR MIH OR HSPM OR Developmental Defects of Enamel[MeSH Terms] OR dentin dysplasia[MeSH Terms] OR Dentinogenesis imperfecta[MeSH Terms] OR Hypophosphatemia, X Linked[MeSH Terms] OR dentin disorder) AND (Dental bonding[MeSH Terms] OR bond* OR etch*)
PMC-PubMed From 1993 to 2024	((Molar AND (Hypomineralization OR Hypomineralized)) OR MIH OR HSPM OR Developmental Defects of Enamel[MeSH Terms] OR dentin dysplasia[MeSH Terms] OR Dentinogenesis imperfecta[MeSH Terms] OR Hypophosphatemia, X Linked[MeSH Terms] OR “dentin disorder“) AND (Dental bonding[MeSH Terms] OR bond* OR etch*) AND ("author manuscript"[Filter]
Web of Science From 1993 to 2024 *Filter 1.49 «* *Dentistry & Oral Medicine* *»*	(Molar AND (Hypomineralization OR Hypomineralized) OR MIH OR HSPM OR “amelogenesis imperfecta” OR “dentinogenesis imperfecta” OR “osteogenesis imperfecta” OR hypophosphatemia OR "dentin dysplasia" OR hypomineralization) AND ("dental bonding" OR bond* OR etch*)

A manual search through the reference lists of the selected studies was conducted. The two reviewers did the data extraction using Zotero, a personal research assistant tool^40^. Any discrepancies were resolved through discussion, and a consensus was reached. Duplicate studies found in multiple databases were eliminated. Two reviewers independently screened the titles and abstracts of the identified studies to determine their eligibility, then compared their selection. Reports not included in the analysis comprised those without SR or SBS tests, those not in English, animal studies, documents not in research article format, and studies unrelated to enamel or dentin defects. Meta-analyses and systematic reviews were also excluded. The extracted information from each eligible study included diagnosis, type of tissue (E/D), teeth (PD/DD), sample size, type of test (SBS/SR), used materials, bonding protocol and numerical values of SBS and SR.

### Evaluation of the level of evidence

The GRADE (Grading of Recommendations Assessment, Development and Evaluation) system was used to grade the certainty of evidence of each outcome^41^. Certainty of evidence consists of confidence in the effect estimates of studies. 4 levels are used to characterize certainty of evidence: high, moderate, low and very low (summarized in Table 3). Randomized controlled trials start at “high” and observational studies at “low”. We then lowered the certainty of evidence based on 5 factors: risk of bias, indirectness (PICO—population, intervention, comparison, outcome—is detailed), imprecision (sample size), inconsistency, and publication bias. Finally, then raise it with size of effect (based on SR or SBS) if it is very large or large. We combined the SBS values for different disease stages and adhesive systems. We employed an average to calculate an SBS value specific to the article’s pretreatment. We opted for a rate for the SBS and an absolute value for the SR. Strength of recommendations reflects the confidence that the authors have that the desirable effects of a management strategy outweigh the undesirable effects. The authors reached an agreement through discussion and attained a consensus. The certainty of evidence was then interpreted as two grades of recommendations: “strong” and “weak”.

**Table 3:** GRADE levels and strength of recommendation^41^.

Certainty of evidence	High	Further research is very unlikely to change our confidence in the estimate of effect.
	Moderate	Further research is likely to have an important impact on our confidence in the estimate of effect and may change the estimate.
	Low	Further research is very likely to have an important impact on our confidence in the estimate of effect and is likely to change the estimate.
	Very Low	Any estimate of effect is very uncertain.
Recommendation	Strong recommendation	Benefits clearly outweigh harms; most patients would want receive this type of intervention.
	Consideration	Some uncertainty about the balance of benefits and harms, clinicians would only refer the intervention under specific conditions.

## Results

**Fig. 1:**
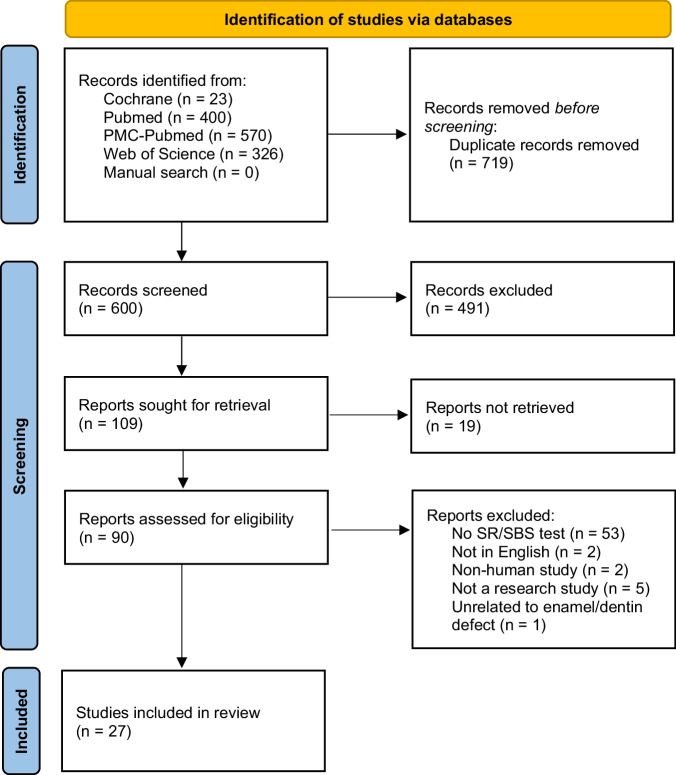
Flowchart of studies selection according to PRISMA statement. SBS Shear Bond Strength or Microtensile Bond Strength, SR Survival rate.

Among the 600 articles obtained after removing duplicates, 109 were considered for a full review after evaluating the title and abstract. As depicted in the flowchart (Fig. 1), studies written in another language than English (n = 2), those not fully available (n = 19), published in a form other than a scientific article (n = 5), those not involving human subjects (n = 2) or not related to the selected pathologies in this study (n = 1) were excluded. Ultimately, 27 studies were selected and included.

The distribution of different types of articles is provided in Table 4. The results exhibit significant heterogeneity. 15 articles address fluorosis: 1 is an in vivo study (enamel & PD), and 14 articles are in vitro studies (12 on enamel & PD, and 2 on dentin & PD). 7 articles focus on MIH: 3 are in vivo studies (3 articles on enamel & PD), and 4 articles are in vitro studies (3 on enamel & PD, 1 on enamel and dentin & PD). 5 studies pertain to AI: all are in vitro (1 article on enamel and dentin & DD, 1 article on dentin & DD, 1 article on enamel & PD, 1 article on enamel and dentin & PD, and 1 article on dentin & PD). No selected article targets HSPM, DI, OI, or XLH.

**Table 4:** Studies repartition.

F	*In vivo*: n = 1	E (PD): n = 1
	*In vitro*: n = 14	E (PD): n = 12 D (PD): n = 2
MIH	*In vivo*: n = 3	E (PD): n = 3
	*In vitro*: n = 4	E (PD): n = 3
		E & D (PD): n = 1
AI	*In vivo*: n = 0	
	*In vitro*: n = 5	E & D (DD): n = 1 D (DD): n = 1 E (PD): n = 1 E & D (PD): n = 1 D (PD): n = 1
HPSM, DI, OI, XLH	n = 0	

*AI* amelogenesis imperfecta, *D* dentin, *DD* deciduous dentition, *DI* dentinogenesis imperfecta, *E* enamel, *F* fluorosis, *HSPM* hypomineralized second primary molars, *OI* osteogenesis imperfecta, *PD* permanent dentition, *XLH* X-linked hypophosphatemia.

The characteristics and results of the 27 articles included in this study have been summarized in Table 5 in order of diagnosis and publication date^34–37,42–64^. The collected data are highly heterogeneous depending on the conditions considered, with no identical protocol found more than once.

**Table 5:** 27 included studies.

Reference	Diag • Tissue ¤ Tooth (Aff/So)	Study (Test)	Materials: • adhesive (SE/ER), others	Bonding protocol	SBS (Mpa)/SR of affected tooth
**Weerasinghe et al.**^42^	F (Mild, Moderate & Severe) • E *¤* PD (60/20)	*In vitro* (SBS)	• Clearfil SE Bond (SE) Primer: Clearfil SE-Bond Primer PhA: K-Etchant	**1:** Etching & Primer & SE **2:** Primer & SE	**↗: Etching before SE** Mild: 32/26 Moderate: 35/30 Severe: 30/25 ↗ *CF for etching before SE*
Ermis et al. ^43^	F (Severe) • E *¤* PD (10/11)	*In vitro* (SBS)	• OptiBond FL (ER) • Clearfil Protect Bond (SE) PhA: *unknown*	**1:** ER **2:** SE *Grinding/no grinding of E*	**= SE and ER** Ground E: 42/45 Unground E: 27/16
Waidyasekera et al.^36^	F (Mild & Moderate) • D *¤* PD (32/16)	*In vitro* (SBS)	• Clearfil Tri S Bond (ER) • Single Bond (SE1) • Clearfil SE Bond (SE2)	**1:** ER **2:** SE1 **3:** SE2	**↗: SE** Mild: 20/29/41 Moderate: 17/24/27 *No AF for SE2*
Noble et al.^44^	F (Severe) • E *¤* PD (52/0)	*In vivo* (SR)	PhA: 37% Primer: Scotchbond Multipurpose Primer Pre-coated brackets: 3M Unitek Victory	**1:** MA & Etching & Primer **2:** Etching & Primer	**= MA/no MA before bonding** SR at 9m: 98%/100%
Ertuğrul et al.^45^	F (Moderate) • E *¤* PD (36/36)	*In vitro* (SBS)	• Peak LC Bond (SE1) • Xeno V (SE2) PhA: UltraEtch Primer: Peak SE Primer	**1:** Etching 15s & SE1 **2:** SE1 **3:** SE2	**↗: ER** 17/13/11 ↗ *CF for ER*
Isci et al.^46^	F (Mild) • E *¤* PD (40/40)	*In vitro* (SBS)	• Transbond XT (SE) PhA: 37% Primer: Transbond Plus Self Etching Primer	**1:** Etching & SE **2:** Primer & SE	**↗: Etching before SE** 15/9
Torres-Gallegos et al.^47^	F (Mild, Moderate & Severe) • E *¤* PD (90/30)	*In vitro* (SBS)	• Excite (ER) • Adper Prompt L-Pop (SE1) • AdheSE One (SE2) PhA: 37%	**1:** ER **2:** SE1 **3:** SE2	**↗: SE1** Mild: 13/12/7 Moderate: 11/14/6 Severe: 9/13/7
Silva-Benítez et al.^48^	F (Moderate & Severe) • E *¤* PD (120/20)	*In vitro* (SBS)	• Transbond XT (ER) PhA: Total Etch	**1:** ER (etching 15s) **2:** ER (etching 150s) **3:** MA & ER (etching 15s)	Moderate F: **↗: Longer etching** 16/19/13 Severe F: **↗: MA before ER** 11/10/13
Shafiei et al.^49^	F (Moderate) • E *¤* PD (70/0)	*In vitro* (SBS)	• Adper Single Bond (ER) • Clearfil SE Bond (SE) Laser: Er,Cr:YSGG PhA: 37%	**1:** ER (etching 20s) **2:** ER (etching 40s) **3:** SE **4:** Etching 30s & SE *Laser-etching/bur-grinding before* *2. not done with bur-grinding*	**No superiority of laser** Laser: 26/29/17/19 Bur: 40/–/32/36
Bakhadher et al.^50^	F (Moderate & Severe) • E *¤* PD (45/45)	*In vitro* (SBS)	• Transbond XT (ER) PhA: Total Etch	**1:** MA & ER **2:** Etching 30s & ER **3:** MA & Etching 30s & ER	**↗: MA & Etching before ER** 4/8/9
Nalçaci et al.^51^	F (Moderate) • E *¤* PD (34/34)	*In vitro* (SBS)	• Transbond XT (ER) PhA: ScotchBond Laser: Er: YAG	**1:** Etching 30s & ER **2:** Laser-etching & ER	**↘: Laser-etching before ER** 15 /12 ↗ *CF with Etching*
Gu et al.^34^	F (Mild, Moderate & Severe) • E *¤* PD (90/30)	*In vitro* (SBS)	• Adper Single Bond 2 (ER) PhA: 35/40/45%	**1:** 35% PhA **2:** 40% PhA **3:** 45% PhA *Etching 30s before bonding*.	**↗: 40% PhA** Mild: 13/15/9 Moderate: 10 /13/8 Severe: 6/10/6
Tan et al.^52^	F (Mild, Moderate & Severe) • D *¤* PD (132/44)	*In vitro* (SBS)	• Prime and Bond NT (ER) • RelyX U200 SE (SE) • RelyX Luting SE (GIC)	**1:** SE **2:** ER & SE **3:** GIC **4:** ER & GIC *Etching 15s for ER*.	**↗: ER & SE** Mild: 4/6/2/3 Moderate: 4/6/1/1 Severe: 3/5/1/1 ↗ *CF with ER*
Cardenas et al.^53^	F (Moderate) • E *¤* PD (49/49)	*In vitro* (SBS)	• Clearfil Universal Bond (SE1) • Futurabond U (SE2) • iBond Universal (SE3) • Scotchbond universal (SE4)	*Each adhesive was applied by 3 ways:* **1:** Etching & SE **2:** Active SE (20s) **3:** Passive SE (20s) *“Active” meaning manual pressure*	**↗: Etching before SE** SE1: 16/13/10 SE2: 14/13/9 SE3: 12/11/9 SE4: 15/15/12
Zhengfan et al.^54^	F (Mild & Moderate) • E *¤* PD (48/0)	*In vitro* (SBS)	• Single Bond Universal (SE) NaOCl: 5.25% PhA: 35%	**1:** Etching & SE **2:** NaOCl (60s) & Etching & SE **3:** NaOCl (120s) & Etching & SE **4:** NaOCl (180s) & Etching & SE	**↗: 60/120s NaOCl before etching** Mild: 26/28/29/23 Moderate: 25/28/31/25 ↗ *CF with NaOCl time*
William et al.^55^	MIH • E ¤ PD (45/44)	In vitro (SBS)	• 3M ESPE Single Bond (SE1) • Clearfil SE Bond (SE2)	1: SE1 2: SE2	**No superiority of SE1 or SE2** 7/10
Chay et al.^56^	MIH • E ¤ PD (84/21)	In vitro (SBS)	• Clearfil SE Bond (SE) NaOCl: 5.25% PhA: 35% Infiltrant: Icon	1: SE 2: Icon & SE 3: NaOCl 60s & Icon & SE 4: NaOCl 60s & SE *Etching 15s at the start*	**↗: 60s NaOCl after etching** 22/19/26/25
Ekambaram et al.^57^	MIH • E ¤ PD (30/30)	In vitro (SBS)	• Adper Single Bond 2 (ER) NaOCl: 5% PhA: 32% Papacarie gel	1: ER 2: NaOCl (60s) & ER 3: Papacarie gel (60s) & ER *Etching 10s at the start*	**↗: 60s NaOCl/papacarie gel after etching** 19/24/25 ↗ CF with NaOCl or papacarie gel
Sönmez et al.^37^	MIH • E ¤ PD (95/31)	*In vivo* (SR)	• Futurabond NR (SE) PhA: Etch-37	**1:** Aff E removed **2:** Aff E ground **3:** Aff E ground & NaOCl (60s) after etching **4:** control	**↗: NaOCl after etching** SR at 24m: 81%/58%/78%/87%
Krämer et al.^58^	MIH • E/D *¤* PD (53/41)	*In vitro* (SBS)	• OptiBond FL (ER) • Clearfil SE Bond (SE1) • Scotchbond Universal (SE2) NaOCl: 5% Infiltrant: Icon	**1:** ER **2:** NaOCl (60s) & ER **3:** NaOCl (60s) & Icon & ER **4:** SE1 **5:** SE2 *2., 3. & 5. were only on E*	Enamel: **↗: NaOCl & Icon before ER** 21/24/25/11/17 Dentin: **↗: ER** 57/–/–/43/–
Rolim et al.^59^	MIH • E *¤* PD (64/0)	*In vivo* (SR)	• Ambar Universal (SE) PhA: Ultra-etch	**1:** Etching & SE **2:** SE *Etching: 30s E, 15s D*.	**↗: Etching before SE** SR at 12m: 81%/62%
Özgür et al.^60^	MIH • E *¤* PD (100/0)	*In vivo* (SR)	• Conceal F (ER) • Beautisealant (SE) PhA: i-GEL N Primer: Beautisealant Primer	**1:** ER **2:** Primer & SE	**↗: ER** SR at 12m: 68%/8%
Saroğlu et al.^61^	AI (Hypocal.) • E/D *¤* DD (7/7)	*In vitro* (SBS)	• Gluma One Bond (ER) PhA: 20% NaOCl: 5%	**1:** Etching (20s) & ER **2:** Etching (20s) & NaOCl (60s) & ER	Enamel: **↗: NaOCl after etching** 14/27 Dentin: **= NaOCl/no NaOCl** 10/9
Hiraishi et al.^62^	AI • D *¤* DD (6/6)	*In vitro* (SBS)	• Prime and Bond NT (ER) PhA: Caulk Tooth Conditionner Gel (34%)	*Etching before P&B* **1:** 15s **2:** 30s	**= 15/30s etching time** 20/21
Faria-e-Silva et al.^63^	AI (Hypocal.) • E/D *¤* PD (5/5)	*In vitro* (SBS)	• Single Bond 2 (ER) NaOCl: 5%	**1:** NaOCl (60s) & ER **2:** ER	Both Enamel & Dentin: **= NaOCl/no NaOCl** Data not shared
Yaman et al.^35^	AI (Hypopla.) • E *¤* PD (18/14)	*In vitro* (SBS)	• Adper single Bond 2 (ER) • Clearfil SE Bond (SE) PhA: 35%	**1:** ER **2:** SE	**= both ER & SE** 20/18
Epasinghe et al.^64^	AI (Hypocal.) • D *¤* PD (4/4)	*In vitro* (SBS)	• Clearfil SE Bond PhA: Caulk Tooth Conditionner Gel (34%)	**1:** Etching (15s) & SE **2:** SE	**↗: Etching before SE** 25/19

*Aff* affected, *AI* amelogenesis imperfecta, *CF* cohesive fracture, *D* dentin, *DD* Deciduous Dentition, *Diag* Diagnostic, *E* enamel, *ER* Etch-and-rinse, *F* fluorosis, *GIC* glass ionomer cement, *HSPM* hypomineralized second primary molars, *m* month, *MA* micro-abrasion, *MIH* molar-incisive hypomineralization, *Mod* moderate, *NaOCl* sodium hypochlorite, *PD* permanent dentition, *PhA* phosphoric acid, *P&B* prime and bond, *SE* self-etch, *SBS* shear bond strength (including microtensile bond strength), *So* sound, *SR* survival rate, *Sev* severe, *%* concentration.

This resulted in the identification of 12 clinical pretreatments of enamel or dentin: 7 for fluorosis, 2 for MIH and 3 for AI. The GRADE levels of evidence “high” and “very high” were translated into strong recommendations that should be followed by practitioners, taking into consideration the size of effect; a large or very large size of effect was needed to achieve a “strong recommendation.” On the other hand, the GRADE levels of evidence “low” and “very low” were translated into considerations. Although the evidence is insufficient for a firm recommendation, these considerations can be useful in certain clinical settings, taking into account the practitioner’s viewpoint and experience within the framework of evidence-based dentistry. Among the 12 pretreatments, we identified 3 strong recommendations and 9 considerations. As there was no study collected on dentinogenesis imperfecta, osteogenesis imperfecta and X-linked hypophosphatemia, there is no recommendation nor consideration for dentin disorders.

The details regarding the assessment of certainty, effect size, GRADE level of evidence, and strength of recommendation are available in the Supplementary Information.

### Recommendations and considerations for bonding in fluorosis

#### Recommendation 1: Bonding on fluorotic enamel should be performed using an etch-and-rinse technique (strong recommendation; very high level of evidence)

In the literature, the ER technique entails using a conditioner to remove dentin debris (most commonly 37% phosphoric acid), followed by a primer and finally the adhesive resin^65^. Self-etch adhesives are made of functional acidic monomers that create a chemical bond between with dental substrates^66^. They directly interact with the dental surface through dentin debris^32,67^. In this study, we define the “etch-and-rinse technique” as the application of acid etching before the adhesive is applied (either standard or self-etch), regardless of whether the manufacturer’s instructions are followed.

There is a very high level of evidence that using an etch-and-rinse technique significantly improves the SBS (+10% to +69%)^42,43,45,46,49,53^, as underscored by six high-quality in vitro studies (number of analyzed teeth = 72 + 80 + 98 + 80 + 70 + 21). Ertuğrul et al. compare two SE adhesives in moderate fluorosis (after etching with 35% phosphoric acid for 15 s, or not), finding both the SBS results and the number of cohesive fractures to be higher in the ER technique group^45^. The work of Isci et al. involves applying phosphoric acid etching for 30 s before the SE, compared to a primer, with etching yielding superior results in moderate fluorosis^46^. Cardenas et al. assess four different SE adhesives, applying them by three ways in moderate fluorosis (with an etching before, with an active application, or with a passive application), showing the ER technique to be more effective for three adhesives^53^. However, an active application of the SE—applying a 35 g pressure with microbrush—resulted in better bonding than a passive application. By Weerasinghe et al. compare the ER technique with the SE technique using a SE adhesive joined to a primer, noting that the ER technique produced higher SBS and cohesive fractures for each fluorosis severity^42^. Shafiei et al. tested the effectiveness of laser to optimize bonding in moderate fluorosis (see recommendation 5), finding that the ER technique exhibits superior SBS, regardless of the enamel preparation type (laser or bur)^49^. In their investigation of severe fluorosis, Ermis et al. observe no significant difference between the techniques, though numerical values lean towards the ER technique^43^. Nonetheless, they highlight the necessity of grinding fluorotic enamel before bonding.

Two in vitro studies (number of analyzed teeth = 120 + 48) report a decrease in SBS (–15% to –39%)^36,47^, presenting a contrasting viewpoint. Among these, the study by Waidyasekera et al., which carries low certainty, mainly concludes that the Clearfil SE Bond adhesive outperforms Single Bond (SE) and Clearfil Tri S Bond (ER) in mild to moderate fluorosis. The second study, by Torres-Gallegos et al., also comparing three adhesives (2 SE, 1 ER), finds primarily that the SE Adper Prompt L-Pop is superior to the other two^47^. However, these studies alone do not undermine the strength of the recommendation, particularly since they focus on comparing different adhesives rather than directly contrasting the two techniques.

The greater volume of studies related to fluorosis can be attributed to its higher prevalence (see Table 1). Nonetheless, there is a notable shortage of in vivo studies to supplement these findings.

#### Recommendation 2: bonding on fluorotic enamel should not be performed after a laser etching (strong recommendation; high level of evidence)

The laser was described during the 1990s by Pick and Colvard as a tool for soft tissue surgeries, and it is also used for water evaporation in hard tissues^68^. Concurrently, numerous studies have indicated that optimal bonding is achieved on dry surfaces, highlighting the potential utility of lasers in attaining this condition. Additionally, some authors have suggested that lasers could serve as a substitute for etching or be utilized for grinding.

There is a high level of evidence that using laser results in a moderate decrease in SBS (–20% to –41%)^49,51^, based on two in vitro studies presenting a low evidence level. Nalçaci et al. found that laser etching with Erbium: YAG for 30 s before applying an ER adhesive yielded inferior outcomes compared to 30 s of acid-etching. Similarly, Shafiei et al. identified the highest performance in groups that underwent conventional grinding with a diamond bur, concluding that the laser does not offer any superiority^49^.

This recommendation may be formulated because laser etching induces excessive dryness, that which hinders the achievement of effective bonding.

#### Considerations

##### A 40% phosphoric acid etching might be better than other concentrations for bonding on fluorotic enamel (high level of evidence)

Some authors hypothesize that increasing the concentration of phosphoric acid would allow better penetration of the acid into tissues, thus leading to improved SBS. However, the most commonly encountered concentration in protocols is 37% phosphoric acid.

There is a high level of evidence that the use of 40% phosphoric acid markedly enhances µTBS (+31%)^34^, drawing from a high-quality in vitro study (number of analyzed teeth = 120). In their research, Gu et al. showed that employing 40% phosphoric acid (instead of 35% and 45%) for 30 s improves SBS on enamel for all severities of fluorosis^34^. The authors attribute this improvement to the observation of a resin-penetrated matrix under the microscope during the bonding interface formation with the 40% etching. Despite the high level of evidence, the study lacked a comparison group for 37% phosphoric acid, the concentration most frequently mentioned in the selected articles. Assuming that 37% closely ~40% significantly dilutes the recommendation’s robustness.

Additional research is needed to expand upon these results.

##### A deproteinization step with 5% NaOCl, for 60 s before etching, might improve the bonding on fluorotic enamel

Sodium hypochlorite is a solution commonly used in endodontics to remove entangled dentin debris from the canals during root canal treatment. It neutralizes amino acids in tissues, resulting in the formation of water and salt, hence the principle of deproteinization^69^. According to the work of Sabandal et al., hypomineralized tissues have a higher amount of proteins on their surface, which poses challenges for the durability of bonding^70^. Pretreatment with 5% NaOCl—a concentration higher than that typically used in endodontics—could potentially reduce the protein load on their surfaces, thereby optimizing the SBS.

There is low-level evidence that the addition of 5% NaOCl in fluorosis moderately improves the SBS (+18%)^54^. The in vitro study on permanent enamel (number of analyzed teeth = 48) by Zhengfan et al. shows that the application of 5.25% NaOCl for 60 and 120 s before etching improves bond strength^54^. The incidence of cohesive fractures rises with longer application times. However, the study notes that excessive deproteinization time (180 s) significantly decreases the surface protein quantity, adversely affecting bonding. For clarity in recommendations and considerations, we use a 5% NaOCl concentration, even though Zhengfan’s study specifies 5.25%. Commercially, the concentration varies between 5% and 6%, and to date, no optimal concentration for tissue deproteinization has yet been established.

##### A longer etching time might improve the bonding on fluorotic enamel (low level of evidence)

Some authors, such as Barkmeier et al. have examined the effectiveness of increasing etching time on bonding and have indicated that it does not have a significant impact on the SBS on healthy enamel^71^. Surface geometry is not substantially altered. However, since surface properties vary on hypomineralized tissues, investigations have been conducted in fluorosis to test this pretreatment.

There is a very low level of evidence that extending the etching time enhances SBS (+7%)^48^. The in vitro study (number of analyzed teeth = 140) by Silva-Benitez et al. finds that bonding improves when the etching duration with 37% phosphoric acid is extended to 150 s instead of 15 s in moderate fluorosis^48^. They propose that the acid achieve deeper penetration and generates a surface roughness similar to that of healthy enamel etched for 15 s. However, the findings indicate that a prolonged etching duration does not yield better results in severe fluorosis. Torres-Gallegos et al. support this observation by noting that lengthening the etching time in severe fluorosis might decrease surface roughness, resulting in a completely intact surface, which could negatively affect retention^72^.

The in vitro studies by Zavala-Alonso and Al-Sugair were excluded from our analysis as they did not assess SBS^73,74^. Nonetheless, they show that the microstructural depth increases by up to 5 µm in moderate fluorosis after extending the etching time. This could potentially enlarge the available bonding surface, thereby enhancing the SBS. Finally, it is worth considering an increase in etching time, although caution is advised due to the low level of evidence supporting this approach. While an increase of 150 s appears excessive in a clinical context, doubling the recommended etching time (60 s on enamel instead of the manufacturers’ instructions of 30 s) may be a viable alternative. However, further research is needed to confirm its effectiveness.

##### A micro-abrasion might improve the bonding on fluorotic enamel (very low level of evidence)

Micro-abrasion, introduced by Croll in the 1990s, entails the superficial removal of discolorations resulting from demineralization on teeth^75^. It is highly recommended for improving bonding to sound tissues^76^.

There is a very low level of evidence indicating that micro-abrasion marginally improves the SBS in fluorosis (+13%)^50^, based on a low-quality in vitro study (number of analyzed teeth = 90) by Bakhader et al. The authors demonstrate that combining microabrasion with etching provides better outcomes than etching alone. However, the values are significantly lower when microabrasion is used instead of etching, underscoring its importance. Two other low-quality studies (n = 52 + 140) indicate that micro-abrasion has minimal impact on the SR at 9 months (–2%) and the SBS (–4%)^44,48^. In the in vivo study by Noble et al., the lack of clear explanation for randomization diminishes its quality. The authors report that SR outcomes are nearly the same at 9 months for groups with and without microabrasion, with only one restoration in the microabrasion group detaching after 4 days. Given the poor quality of this study, it carries little weight in this recommendation. The study by Silva-Benitez et al. finds that microabrasion slightly reduces the SBS in moderate fluorosis but enhances it in severe fluorosis.

These results, while not highly significant, should not discourage practitioners from incorporating microabrasion into their bonding procedures for fluorosis. This is due to its proven effectiveness in bonding to healthy enamel and the low quality of the studies underpinning this recommendation.

##### A succession of etch-and-rinse adhesive and a self-etch adhesive might improve the bonding on fluorotic dentin (low level of evidence)

The ER and SE techniques do not have the same effect on tissues (see recommendation 2). Some authors have mentioned that a combination of these two techniques could enhance the benefits of each in the same protocol.

There is low-level evidence that the succession of ER adhesive and SE adhesive significantly increases SBS (+55%)^52^, according to a low-quality in vitro study by Tan et al. on permanent teeth (number of analyzed teeth = 176). The authors found that implementing the technique before applying a SE or glass-ionomer cement (GIC) enhances the SBS. The incidence of cohesive fractures rises with the inclusion of the ER technique. However, the study’s lack of clear randomization significantly diminishes its quality and, by extension, the reliability of this recommendation.

This aligns with Recommendation 1 and, through deduction, further supports the preference for an ER technique over an SE technique.

### Recommendations and considerations for bonding on MIH

#### Recommendation 3: Bonding on MIH enamel should be performed using an etch-and-rinse technique (strong recommendation; high level of evidence)

This recommendation is based on the same thinking as the Recommendation 2.

There is a moderate level of evidence that using an ER technique significantly improves the SBS (+50%)^58^ and the SR (+19% to +60%)^59,60^. This recommendation is supported by three studies on PD (number of analyzed teeth = 94 + 100 + 64), comprising two high-quality studies and a lower-quality one. The in vitro study by Krämer et al. was performed on previously extracted human permanent teeth. The authors first compared ER and SE technique, using 3 different adhesives. In a subsequent phase, they assess the SBS with or without the addition of NaOCl with or without an ICON infiltration (30 s) as it is mentioned in Recommendation 1. The results suggest the superiority of a meticulously performed ER technique on enamel, and that the addition of NaOCl and ICON further enhances bonding. Concurrently, the authors determine that the ER technique also yields better results on dentin. The in vivo study by Rolim et al. investigates the SR over 12 months with a SE adhesive, assessing the impact of acid etching applied for 30  on the enamel^59^. This approach is shown to be significantly more effective than applying the SE adhesive alone. The study by Özgür et al. evaluate a “giomer” adhesive, which failed to demonstrate strong bonding efficacy^60^. The SR is significantly higher for the group that received the ER technique, although the results must be considered with caution due to the ineffectiveness of the selected SE.

In MIH, increased fragility of hypomineralized tissues requires a higher amount of material to be removed before bonding^70^. The SE adhesive’s action might not be as effective as phosphoric acid in the ER technique, despite simplifying the procedure for the practitioner. Given that the selected studies primarily focus on enamel, this recommendation mainly pertains to enamel. Further reasearch is needed to extend it to dentin. This recommendation is supported by the work of Alvarez et al., which finds the SBS on MIH to be superior with an ER technique compared to an SE^3^. However, the systematic review by Lagarde et al. indicates that there is no significant difference in bonding in MIH between the two systems^77^.

#### Consideration

##### A deproteinization step with 5% NaOCl for 60 s might improve the bonding on MIH enamel (moderate level of evidence)

This consideration is based on the same thinking than its equivalent for fluorotic enamel.

There is a moderate level of evidence that deproteinization of enamel with NaOCl marginally enhances the SR and the SBS. This consideration draws from four studies (number of analyzed teeth = 126 + 94 + 60 + 105). An in vivo study by Sönmez et al. shows that the addition of 5% NaOCl for 60 s after etching on MIH-affected teeth increases the SR after 24 months (+20%)^37^. An in vitro study by Krämer et al. highlights the effectiveness of enamel bonding when 5% NaOCl is applied for 60 s before etching (+14% of µTBS)^58^. Moreover, the SBS can be increased when this step is followed by an ICON infiltration. Outcomes for these parameters are not provided for dentin. Ekambaram and Chay studies confirm the efficacy of NaOCl applied for 60 s after etching, enhancing the SBS by 44%^57^ and 16%^56^.

This consideration is consistent with the systematic review of Lagarde et al. They note that bonding is not significantly different based on the type of adhesive technique (SE or ER), but is improved by deproteinization (5% NaOCl for 60 s) after etching in MIH^77^. However, this pretreatment has not been identified as a recommendation due to the limited research available to corroborate the findings.

### Considerations for amelogenesis imperfecta

#### A deproteinization step with 5% NaOCl for 60 s might improve the bonding on AI enamel (very low level of evidence)

This consideration is based on the same thinking than its equivalent for fluorotic enamel.

There is a very low level of evidence that adding 5% NaOCl significantly increases SBS (+93%) on enamel affected by AI, based on two very low-quality in vitro studies (number of analyzed teeth = 14 + 10)^61,63^. Saroğlu et al.’s study on primary teeth illustrates that applying 5% NaOCl (60 s) after etching with phosphoric acid for 20 s significantly increases the SBS for enamel, a result not mirrored in dentin, where SBS with or without NaOCl does not differ significantly. Faria-e-Silva et al.’s study on permanent teeth indicates that deproteinization with 5% NaOCl for 60 s before etching does not significantly affect SBS for either enamel or dentin, though the absence of disclosed numerical SBS values calls for a nuanced interpretation^63^. This is further explored by Ahmed et al.’s study on the enamel of primary teeth (number of analyzed teeth = 10), which adds NaOCl before etching^78^, focusing not on SBS but on the depth of etching penetration after 15 s of application, finding an increase for the NaOCl group compared to controls.

The in vitro studies by Pugach et al. were not included in this analysis as they were not conducted on human subjects^79,80^. These studies were carried out on permanent teeth extracted from mice with AI. The 2014 study examined the effects of deproteinization with 5% NaOCl before applying a SE technique. The authors found no significant difference in SBS between the control group and the test group for both affected enamel and dentin.

Further research involving SBS and SR tests is essential to reinforce this consideration. The findings from these articles provide limited insights on dentin, preventing a definitive recommendation concerning its pretreatment.

#### An etch-and-rinse technique might improve bonding on AI enamel and dentin (low level of evidence)

This consideration is based on the same thinking as the Recommendation 1.

There is a low level of evidence that using an ER technique increases SBS (+11 to +32%)^35,64^, supported by two low-quality in vitro studies (number of analyzed teeth = 8 + 32). The study by Epasinghe et al. demonstrates that etching for 15 s before applying SE increases SBS in hypocalcified AI-affected dentin. Yaman et al. indicate that the µTBS for groups undergoing etching with 35% phosphoric acid (30 s) followed by a two-layer adhesive application differs slightly from groups using a SE technique. Although the increase in SBS between the two test groups is minimal, it still leans in favor of the ER technique, thus supporting this consideration. As noted in the previous recommendation, the 2011 study by Pugach et al. on animal subjects showed a greater incidence of cohesive fractures in enamel of teeth treated with an SE technique compared to an ER technique^79^, which is consistent with this consideration. The results cited are not sufficient identify a recommendation; further investigations on humans are needed.

#### A 30 s etching time might improve the bonding on AI dentin (very low level of evidence)

This consideration is based on the same thinking as the consideration of a longer etching time for bonding on fluorotic enamel.

There is weak evidence that increasing the etching time marginally increases SBS (+6%)^62^, stemming from an in vitro study on primary teeth (number of analyzed teeth = 12) by Hiraishi et al. The authors found that increasing the etching duration from 15 to 30 s on affected dentin results in a slight improvement in SBS, whereas bonding decreases on healthy dentin with the 30 s etching. A 34% phosphoric acid etch was used, followed by the application of a two-step adhesive and prime & bond.

This consideration is specific to dentin; and additional investigations are required to extend its application to enamel.

## Discussion

Many patients suffer from systemic diseases that adversely affect the structural integrity of their dental tissues, posing challenges for the bonding of direct restorations. This review illustrates that specific tissue pretreatment techniques can significantly enhance the shear bond strength or survival rate of restorations.

The results of this study vary depending on the type of disease; the majority of the selected research has been conducted in vitro, following the extraction of human teeth. Additional in vivo studies are therefore necessary to confirm these findings. In this study, we assumed that the tissues of deciduous and permanent teeth share identical characteristics. Consequently, the recommendations and considerations do not differentiate between the type of teeth involved. We believe that these pretreatments are not limited to a specific severity of disease, as the results did not vary significantly with the severity of the disease studied. Additionally, the research selected for this analysis primarily addresses hypocalcified AI. There is a clear need for additional studies to broaden these recommendations to encompass all types of AI.

No articles focusing on specific dentin disorders, such as dentinogenesis imperfecta (DI), osteogenesis imperfecta (OI), or X-linked hypophosphatemia (XLH), were incorporated into this study. This stands in contrast to the study by Massé et al.^4^, which is solely concerned with bonding on dentin disorders. This discrepancy may stem from the fact that the 2021 study is largely founded on an analysis of the chemical, mineral, and microstructural properties of tissues for article selection. As such, it is recommended that future research specifically address dentin diseases, including XLH.

This study concentrates on direct bonding but could also extend to the cementation of prosthetic restorations in cases of enamel and dentin disorders. In their systematic review, Strauch and Hahnel propose that indirect restorations should be preferred whenever possible for managing patients with such conditions^81^. Similarly, Broutin et al. mention that indirect restorations are excellent solutions for MIH management^82^.

Finally, this systematic review is based primarily on in vitro studies with a low level of evidence. This results in mostly considerations that did not get identified as recommendations, indicating a significant need for further research on the subject.

## Conclusion

This study allows us to recommend prioritizing bonding on enamel using an etch-and-rinse technique for fluorosis and MIH, supported by a strong recommendation and a very high level of evidence. Other considerations, which have lower levels of evidence, include incorporating a deproteinization step with 5% NaOCl and extending the etching time. However, these should be approached with caution within an evidence-based dentistry framework. Further research, including shear bond strength and survival rate tests, is essential to strengthen these considerations and establish clear recommendations for practitioners.

## Supplementary information

Supplementary Information

PRISMA Checklist

## Data Availability

All data generated or analyzed during this study are included in this published article and its supplementary information files.
