# Supplementary material for: Pretreatments to bonding on enamel and dentin disorders: a systematic review
Source: Evid Based Dent. 2024 Jul 23;25(4):215. doi: 10.1038/s41432-024-01037-z (PMC11661966; doi:10.1038/s41432-024-01037-z)
Supplement: Supplementary file 1 — Supplementary Information [file 41432_2024_1037_MOESM1_ESM.pdf]

Supplementary information

1. Recommendations and considerations for fluorosis

Fluorosis	#1								#2	
Tissue	E	E	E	E	E	E	E	E	E	D
	45	51	49	48	43	46	53	42	47	36
1.**Non-randomization**	**0**	**0**	**0**	**0**	**0**	**0**	**0**	**0**	**0**	**0**
2.Non-indication of severity	0	0	0	0	0	0	0	0	0	0
3.No control group	0	0	0	0	0	0	0	0	0	0
4.*In vitro* study	1	1	1	1	1	1	1	1	1	1
5.Protocol not clearly described	0	0	0	0	0	0	0	0	0	0
Risk of bias (interpretation)	+	+	+	+	+	+	+	+	+	+
PICO detailed	+	+	+	+	+	+	+	+	+	+
Precision (sample size)	+	+	+	+	-	+	+	+	+	-
No publication bias	+								-	
Inconsistency of results	+								+	
**Certainty**	**HIGH**								**LOW**	
Type of value	SBS	SBS	SBS	SBS	SBS	SBS	SBS	SBS	SBS	SBS
Numerical value	↗30%	↘20%	↘41%	↗7%	↗69%	↗67%	↗10%	↗20%	↘15%	↘39%
**Size of effect**	VL	L	VL	VS	VL	VL	S	L	S	VL
**GRADE of evidence quality**	**VERY HIGH**								**HIGH**	
**Recommendation (R) or Consideration (C)**	**R**								**R**	

Size of effect for fluorosis:

> 30% = Very Large (VL)
[20%;30%] = Large (L)
[10%;20%] = Small (S)
< 10% = Very Small (VS)

Fluorosis	#A	#B	#C	#D			#E
Tissue	E	E	E	E	E	E	D
	34	54	49	50	44	48	52
1.**Non-randomization**	**0**	**0**	**0**	**1**	**1**	**0**	**1**
2.Non-indication of severity	0	1	0	0	0	0	0
3.No control group	0	0	0	0	0	0	0
4.*In vitro* study	1	1	1	1	0	1	1
5.Protocol not clearly described	0	0	0	0	0	0	0
Risk of bias (interpretation)	+	+	+	+	+	+	+
PICO detailed	+	+	+	+	+	+	+
Precision (sample size)	+	-	+	+	+	+	+
No publication bias	-	-	-	-			-
Inconsistency of results	NA	NA	NA	-			NA
**Certainty**	**LOW**	**VERY LOW**	**LOW**	**VERY LOW**			**VERY LOW**
Type of value	SBS	SBS	SBS	SBS	SR (9m)	SBS	SBS
Numerical value	↗31%	↗18%	↗19%	↗13%	-2%	↘4%	↗55%
**Size of effect**	VL	S	S	S	VS	VS	VL
**GRADE of evidence quality**	**HIGH**	**VERY LOW**	**LOW**	**VERY LOW**			**LOW**
**Recommendation (R) or Consideration (C)**	**C**	**C**	**C**	**C**			**C**

Size of effect for fluorosis:

> 30% = Very Large (VL)
[20%;30%] = Large (L)
[10%;20%] = Small (S)
< 10% = Very Small (VS)

**OUTCOMES (in Fluorosis)**	**Numer of teeth (Number of studies)**	**GRADE of evidence quality**	**Recommendation or Consideration**
1. Better to use ER technique than SE	n = 72 + 21 + 80 + 98 + 80 + 120 + 48 + 70 (8 studies)	VERY HIGH	Recommendation
2. Laser preparation doesn't increase bonding	n = 68 + 70 (2 studies)	HIGH	Recommendation
A. Using a 40% etching increases bonding	n = 120 (1 study)	HIGH	Consideration
B. Added NaOCl increases bonding	n = 48 (1 study)	LOW	Consideration
C. Longer etching time increases bonding	n = 140 (1 study)	LOW	Consideration
D. MA increases bonding	n = 90 + 52 + 140 (3 studies)	VERY LOW	Consideration
E. Using ER before SE increases bonding	n = 176 (1 study)	LOW	Consideration

2. Recommendations and considerations for MIH

MIH	#3			#F			
Risk of bias:	58	60	59	37	58	57	56
**1.Non-randomization**	**0**	**0**	**0**	**0**	**0**	**0**	**1**
2.Non-indication of severity	1	1	1	1	1	1	1
3.No control group	0	0	0	0	0	0	0
4.*In vitro* study	1	0	0	0	1	1	1
5.Protocol not clearly described	0	0	0	0	0	0	0
Risk of bias (interpretation)	+	+	+	+	+	+	+
PICO detailed	+	+	+	+	+	+	+
Precision (sample size)	+	+	+	+	+	+	+
No publication bias	+			-			
Inconsistency of results	+			+			
**Certainty**	**HIGH**			**MODERATE**			
Type of value	SBS	SR (12m)	SR (12m)	SR (24m)	SBS	SBS	SBS
Numerical value	↗50%	+60%	+19%	+20%	↗14%	↗44%	↗16%
**Size of effect**	**VL**	**VL**	**S**	**L**	**S**	**VL**	**S**
**GRADE of evidence quality**	**HIGH**	**HIGH**	**HIGH**	**MODERATE**			
**Recommendation (R) or Consideration (C)**	**R**	**R**	**R**	**C**			

Size of effect for MIH:
> 30% = Very Large (VL)
[20%;30%] = Large (L)
[10%;20%] = Small (S)
< 10% = Very Small (VS)

**OUTCOMES (in MIH)**	**Number of teeth (Number of studies)**	**GRADE of evidence quality**	**Recommendation or Consideration**
3. Better to use ER technique than SE	n = 94 + 100 + 64 (3 studies)	HIGH	Recommendation
F. Added NaOCl step increases bonding	n = 126 + 94 + 60 + 105 (4 studies)	MODERATE	Consideration

3. Recommendations and considerations for AI

AI	#G		#H		#I
Tissue	E	E	D	E	D
Risk of bias:	[61]	[63]	[64]	[35]	[62]
1.**Non-randomization**	**0**	**0**	**1**	**0**	**0**
2.Non-indication of severity	1	1	1	1	0
3.No control group	0	0	0	0	0
4.*In vitro* study	1	1	1	1	1
5.Protocol not clearly described	0	0	0	0	0
Risk of bias (interpretation)	+	+	-	+	+
PICO detailed	+	+	+	+	+
Precision (sample size)	-	-	-	+	-
No publication bias	-		-		-
Inconsistency of results	-		+		NA
**Certainty**	**VERY LOW**		**LOW**		**VERY LOW**
Type of value	SBS	SBS	SBS	SBS	SBS
Numerical value	↗93%	NA	↗32%	↗11%	↗5%
**Size of effect**	S	VS	VL	S	VS
**GRADE of evidence quality**	**VERY LOW**		**LOW**		**VERY LOW**
**Recommendation (R) or Consideration (C)**	**C**		**C**		**C**

Size of effect for AI:
> 30% = Very Large (VL)
[20%;30%] = Large (L)
[10%;20%] = Small (S)
< 10% = Very Small (VS)

**OUTCOMES (in AI)**	**Number of teeth (Number of studies)**	**GRADE of evidence quality**	**Recommendation or Consideration**
G. Added NaOCl increases bonding for enamel	n = 14 + 10 (2 studies)	VERY LOW	Consideration
H. Better to use ER technique than SE	n = 8 + 140 (2 studies)	LOW	Consideration
I. Longer etching time increases bonding for dentin	n = 12 (1 study)	VERY LOW	Consideration
